# Superfluid density from magnetic penetration depth measurements in Nb–Cu 3D nano-composite films

**DOI:** 10.1038/s41598-020-75351-8

**Published:** 2020-10-27

**Authors:** Chandan Gupta, Pradnya Parab, Sangita Bose

**Affiliations:** School of Physical Sciences, UM-DAE Center for Excellence in Basic Sciences, University of Mumbai, Kalina Campus, Mumbai, 400098 India

**Keywords:** Physics, Condensed-matter physics, Superconducting properties and materials, Surfaces, interfaces and thin films

## Abstract

Superconductivity in 3D Nb–Cu nanocomposite granular films have been studied with varying thickness for two different compositions, Nb rich with 88 at% of Nb and Cu rich with 46 at% of Nb. For both compositions, the superconducting transition temperature (T_c_) decreases with decreasing film thickness. For any thickness, doubling the Cu content in the films decreases the T_c_ by about 2 K. To explore if phase fluctuations play any role in superconductivity in these 3D films, the superfluid stiffness (J_S_) of the films was measured using low frequency two-coil mutual inductance (M) technique. Interestingly, the measurement of M in magnetic fields showed two peaks in the imaginary component of M for both Nb rich and Cu rich films. The two peaks were associated with the pair-breaking effect of the magnetic field on the intra and inter-granular coupling in these films consisting of random network of superconductor (S) and normal metal (N) nano-particles. Furthermore, J_S_ was seen to decrease with decreasing film thickness and increasing Cu content. However, for all films studied J_S_ remained higher than the superconducting energy gap (∆) indicating that phase fluctuations do not play any role in superconductivity in the film thickness and composition range investigated. Our results indicate that an interplay of quantum size effects (QSE) and superconducting proximity effect (SPE) controls the T_c_ with composition in these 3D nano-composite films.

## Introduction

It is well established that the mean field Bardeen–Cooper–Schrieffer (BCS) theory of superconductivity describes superconductors with low superconducting transition temperature (T_c_) quite well^1^. The large Copper pair density leads to a very rigid phase of the condensate wavefunction, even very close to T_c_ suppressing any effect of fluctuations in them. In the past it has been shown that the phase rigidity can be tuned in the low T_c_ superconductors through different ways, primarily in 2D^2–7^. Recently, in 3D films too, with homogenous dis-order, superconducting co-relations was seen to persist even when the global superconductivity of the films was destroyed^8,9^. 2D granular superconductors consisting of small superconducting particles with dis-ordered *insulating* inter-granular regions have also been studied in the past where phase fluctuations of the order parameter was shown to destroy superconductivity^10^. However, the role of phase fluctuations in 3D granular films consisting of random network of superconducting (S) and normal metal (N) nano-particles is largely unexplored. Most results on similar systems studied in earlier times through transport measurements were interpreted from a percolation model^11–14^. Other competing mechanisms have also been invoked to understand superconductivity in such 3D systems above the percolation threshold. Mechanisms like quantum size effects (QSE) arising in the nanoparticles and superconducting proximity effect (SPE) arising from the close proximity of the superconductor and normal metal is also expected to affect superconductivity^15–17^ and both are known to decrease T_c_. In some studies, localization was also reported to affect T_c_ in the films^18^. To understand the predominant mechanism, one needs to measure the superconducting properties by tuning the coupling between the S and N grains. In addition to the conventional superconducting properties like T_c_, critical fields (H_c2_), critical currents (I_c_) etc., one needs to measure the two important energy scales for superconductors i.e. the pair breaking energy, ∆ (superconducting energy gap) and the energy cost to twist the phase, J_S_ (superfluid stiffness) in these films.

∆ can be directly measured through spectroscopic techniques like planar tunneling, scanning tunneling spectroscopy (STM) or point contact Andreev reflection spectroscopy (PCAR). In recent times it has been possible to get an accurate estimation of both ∆ and J_S_ by THz optical spectroscopy^19–22^. However, this technique limits measurements in extremely thin films which need to be transparent to the THz radiation. Another alternative technique which has been successfully used to study different problems in superconductivity in relatively thick superconducting films, is the measurement of penetration depth (λ) using the two-coil mutual inductance technique^23–29^. Thus, in the present work, the 3D nano-composite films will be explored by this technique which is expected to give J_S_ as well as ∆ for different compositions and film thickness.

In this paper, we report the study of the superconducting properties as a function of thickness in Nb–Cu nano-composite granular films with two different compositions: Nb 88 at% (Nb rich) and Nb 46 at% (Cu rich). For both compositions, the superconducting transition temperature, T_c_ decreases with decreasing film thickness as expected. Besides, other superconducting properties was also seen to decrease with an increase in Cu concentration. Interestingly, mutual inductance measurements in magnetic field have shown features associated with intra and inter-granular coupling in these films. The imaginary part of the mutual inductance in magnetic field shows two peaks: The high temperature peak is associated with the pair breaking effect of the magnetic field on the superconductivity of the individual Nb nano-grains. The low temperature peak is related to the breaking of the weak links in the random network of S–N–S (Nb–Cu–Nb) Josephson junctions. Further insight into the inter-granular coupling between the Nb grains in the nanocomposite films was obtained from the measurement of J_S_ by the two-coil mutual inductance method. J_S_ was seen to decrease with reducing film thickness for both the concentrations. Furthermore, with increasing Cu concentration, it was seen to substantially decrease leading to large enhancements in the superconducting penetration depth. However, the low temperature values of J_S_ were about an order of magnitude higher than the superconducting energy gap indicating that in the regime of investigation, phase fluctuations are still not dominant in controlling the superconducting properties for these 3D nano-composite films. Furthermore, we argue that the variation of T_c_ with composition is an interplay of QSE and SPE and it is very difficult to isolate just one effect due to the complicated microstructure. Finally, the present study shows that for phase fluctuations to dominate in 3D films with a random network of SNS junctions, very high Cu concentrations or very low thickness is required, almost close to the 3D-2D cross-over boundary.

## Experimental details

**Figure 1:**
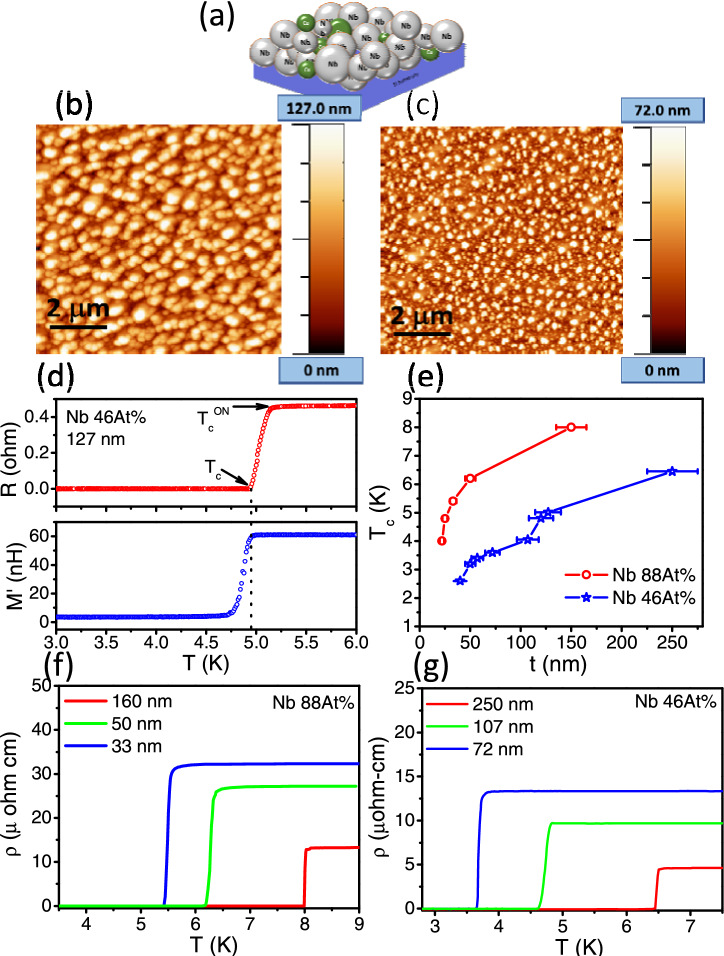
(**a**) Schematic of Nb–Cu nano-composite film morphology. (**b**,**c**) AFM images of two Cu rich films (Nb 46 at%) with thickness ~ 127 nm and ~ 72 nm respectively. (**d**) A representative plot to determine the superconducting transition temperature (T_c_) from Resistance (R) vs temperature (T) (top panel) and mutual inductance (M) vs temperature (T) (bottom panel) measurements. Shown here is the data for a 127 nm thick Cu rich (Nb 46 at%) film. T_c_^ON^ is the onset of the transition in RT measurements and T_c_ is the temperature where R goes to zero and the onset of the diamagnetic shielding response in the film. (**e**) Variation of the transition temprature (T_c_) with thickness (t) for the Nb–Cu films. Red circles denote the variation for the Nb rich (Nb 88 at%) films while the blue stars denote the variation for the Cu rich (Nb 46 at%) films. (**f**) Resistivity (ρ) as a function of temperature (T) for Nb rich (Nb 88 at%) films for three thickness, 160 nm (red), 50 nm (green) and 33 nm (blue). (**g**) Resistivity (ρ) as a function of temperature (T) for Cu rich (Nb 46 at%) films for three thickness, 250 nm (red), 107 nm (green) and 72 nm (blue).

Nb-Cu nanocomposite films were grown by DC and RF co-sputtering of Nb and Cu on Si(100) substrates which had a 100 nm thick native layer of SiO_2_. The substrate temperature during deposition was kept at 600 °C. The composition in the films was controlled by the ratio of the sputtering power of Nb and Cu. The pressure of the Ar gas was kept constant at ~ 5.5 × 10^−3^ mbar for all the films. The thickness was controlled by the time of deposition which was optimized for a given composition. The growth parameters used for the sputtering of the films studied are listed in Table 1. The films were characterized by XRD which showed peaks corresponding to BCC Nb and FCC Cu indicating the formation of nano-crystalline films. Thus, random network of Nb–Cu–Nb (superconductor-normal metal-superconductor or SNS) junctions were formed in the 3D granular films (For a schematic of the sample morphology, see Fig. 1a). The grain size of the Nb and Cu crystallites varied with film composition and thickness, between 16 and 8 nm and 10 to 20 nm, respectively. More details of the growth and characterization of similar but thicker films can be found elsewhere^30,31^. For this work, films of varying thickness were grown with two different compositions of Nb:Cu. The Nb rich films had 88 at% of Nb and the Cu rich films had 46 at% of Nb (will be denoted as Nb 88 at% and Nb 46 at% in this work). The compositions were determined using energy dispersive X-ray analysis (EDAX). The thickness of the films were measured using a surface profilometer and cross-verified using atomic force microscopy (AFM). The morphology and the granular nature of the films were determined using scanning electron microscopy (SEM) and AFM. Figure 1b,c shows the representative AFM images of two Cu rich films with different thicknesses (127 nm and 72 nm, respectively). With decreasing thickness, while films look more or less same with the same granular nature, the crystallites seem to have decreased in size (also observed from X-ray diffraction line broadening), the crystallite density appears to have decreased and the surface roughness of the films also reduces.

**Table 1:** Growth parameters along with the composition (in at%), thickness (in nm) and T_c_ (in K) of the Nb–Cu nanocomposite films.

Samples	Nb:Cu ( at%)	Pressure (mbar)	Power (watt)		Time (s)	Thickness (nm)	T_c_ (K)
			Nb	Cu			
Nb rich	88:12	5.4E−3	179	9.6	300	160	8.0
	88:12	5.4E−3	200	30	45	50	6.2
	88:12	5.4E−3	200	30	37	33	5.4
	88:12	5.5E−3	200	30	30	25	4.8
	88:12	5.5E−3	200	30	20	22	4.0
Cu rich	46:64	5.5E−3	120	150	300	250	6.45
	46:64	5.4E−3	300 (RF)	200	60	164	6.2
	46:64	5.5E−3	188	143	60	127	5.03
	46:64	5.4E−3	300 (RF)	200	35	120	4.8
	46:64	5.5E−3	188	143	45	106	4.05
	46:64	5.5E−3	188	143	30	72	3.5
	46:64	5.5E−3	300 (RF)	200	22	50	3.2
	46:64	5.5E−3	182	142	15	40	2.6

The superconducting transition temperature (T_c_) of the films was measured by dc transport and through two-coil mutual inductance technique. The dc transport was done using the usual four probe method using the Keithley 2400 sourcemeter for which the samples were grown in the Hall geometry using shadow masks. The mutual inductance was measured using the two-coil technique^24,25^ at a low frequency of 30 kHz which was also used to measure the superfluid stiffness (J_S_) and the magnitude of the penetration depth (λ) of the films. For this, the films were grown in a circular geometry of 8 mm diameter, also using a shadow mask. The mutual inductance between the primary and the secondary coil with the film in between was measured as a function of temperature by passing a small ac excitation current ~ 0.5 mA through the primary and measuring the in-phase and out-of-phase induced voltage in the secondary using a lock-in amplifier. The mutual inductance was evaluated for different values of λ by numerically solving the Maxwell and London’s equations which was compared with the experimentally measured value^24,25^. This gave the value of λ for the films. The low temperature measurements (both transport and J_S_) were done in a cryogen free system with a base temperature of 2.7 K. The mutual inductance measurements in magnetic fields were done in an usual bath cryostat with the base temperature of 1.2 K having a maximum magnetic field of 13 T.

## Results and discussions

Figure 1d shows a representative plot (for a Cu rich film with Nb 46 at%) to determine the superconducting transition temperature (T_c_) of the nanocomposite films. The upper panel shows the resistance vs temperature (R–T) where T_c_ is the temperature where the resistance goes below the measurable limit. This corresponds to the onset of the diamagnetic shielding response of the films as measured from the real part of the mutual inductance (M′–T) shown in the bottom panel measured using the two-coil method. The T_c_ of all the films studied in this work is shown for both compositions as a function of film thickness (t) in Fig. 1e. The graph shows two distinct results: First for both compositions (Nb rich and Cu rich), T_c_ decreases with reducing film thickness, a result well known in superconducting thin films^23^. The second result is that with increasing Cu content in the films, T_c_ also decreases (open blue stars in Fig. 1e), such that it decreases to as low as 3.5 K for a relatively thick, 72 nm film with a composition of Nb 46 at%. (Note: Further increasing the Cu content would lower the T_c_ of the films substantially thereby pushing the boundaries of measurements). This result was also obtained in our previous studies on very thick films where the T_c_ was seen to gradually decrease with increasing Cu content^31^. As explained previously, T_c_ is associated with the temperature at which there is global phase coherence in the films. Hence, it is expected that T_c_ should be primarily controlled by the coupling of the Nb grains via the Cu grains in these films. However, with decreasing film thickness, the room temperature resistivity (ρ_N_ at 8 K) increased marginally but remained quite low (~ μohm-cm) implying that the films remain above the percolation threshold down to the lowest thickness probed (see Fig. 1f,g). Besides, the resistivity of the Cu rich films also becomes lower than the Nb rich films due to the large increase in the Cu content. Thus, all films studied here were purely metallic in nature unlike the granular Al films which were in the insulating regime used to probe the role of phase fluctuations on superconductivity^19–22^. From our previous studies on thick films, we had conjectured that phase fluctuations might play a dominant role in controlling the T_c_ with increasing Cu content in these granular Nb–Cu films. However, the question which follows is whether this is possible with the films being so metallic with large inter-granular coupling. To conclusively establish this, measurement of J_S_ needs to be done for these nano-composite films.

**Figure 2:**
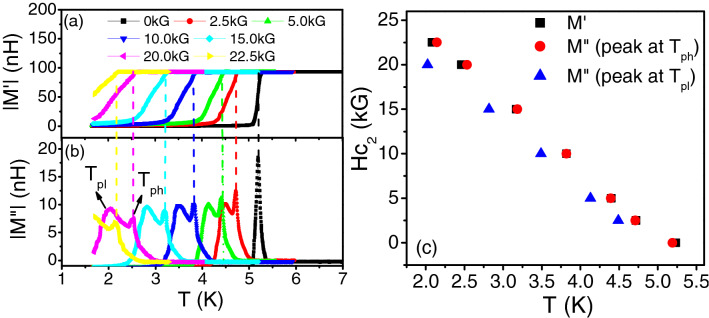
(**a**) Plot of the absolute value of the real part of mutual inductance (|M′|) with temperature (T) for different magnetic fields (denoted in the legend) for a 33 nm thick Nb rich (Nb 88 at%) film. (**b**) Plot of the absolute value of the imaginary part of mutual inductance (|M″|) with temperature (T) for the same magnetic fields as shown in (**a**) and for the same film. The higher temperature peak (T_ph_) is associated with the dissipation arising due to the movement of intra-granular vortices within Nb grains and the peak at lower temperature (T_pl_) is associated with the dissipation arising due to the breaking of inter-granular Josephson coupling. (**c**) Phase diagram (H_c2_ vs T) for the 33 nm thick Nb rich film. The black squares are obtained from (**a**) while the red circles and blue triangles are obtained from the high temperature peaks and low temperature peaks respectively from (**b**).

**Figure 3:**
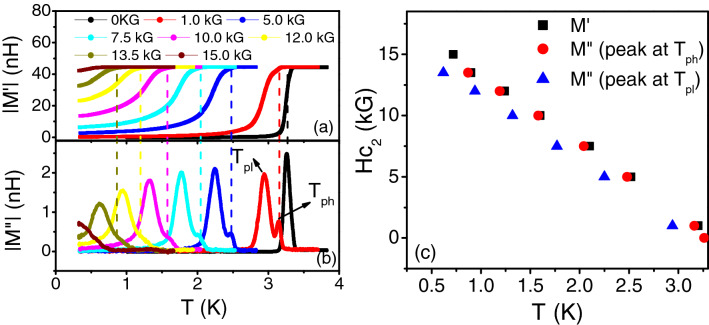
(**a**) Plot of the absolute value of the real part of mutual inductance (|M′|) with temperature (T) for different magnetic fields (denoted in the legend) for a 72 nm thick Cu rich (Nb 46 at%) film. (**b**) Plot of the absolute value of the imaginary part of mutual inductance (|M″|) with temperature (T) for the same magnetic fields as shown in (**a**) and for the same film. The higher temperature peak (T_ph_) is associated with the dissipation arising due to the movement of intra-granular vortices within Nb grains and the peak at lower temperature (T_pl_) is associated with the dissipation arising due to the breaking of inter-granular Josephson coupling. (**c**) Phase diagram (H_c2_ vs T) for the 72 nm thick Cu rich film. The black squares are obtained from (**a**) while the red circles and blue triangles are obtained from the high temperature peaks and low temperature peaks respectively from (**b**).

Now, ${J}_{S}= \frac{{\hbar}^{2}a{n}_{S}}{4{m}^{*}}$ where *a* is the length scale over which the phase fluctuates and can be taken as the superconducting coherence length, ξ, m ^*^ is the effective mass of the electron and n_S_ is the superfluid density given by: ${n}_{S}= \frac{{m}^{*}}{4\pi X{10}^{-7}{e}^{2}}\frac{1}{{\lambda }^{2}}$, where *e* is the electronic charge and λ is the London penetration depth^1,2^. Thus, J_S_ is inversely proportional to the square of λ and the measurement of λ by the two coil mutual inductance measurements for finite dimension films can give J_S_. However, for quantitative estimation, ξ needs to be determined for the films. For this we measured the upper critical fields since H_C2_ is given by: ${H}_{C2}= \frac{{\phi }_{0}}{2\pi {\xi }^{2}}$ where, ϕ_0_ is the flux quantum, 2.07 × 10^−15^ A-m. To measure H_C2_, mutual inductance was measured as a function of temperature for different magnetic fields. Figure 2a,b shows the real and imaginary parts of the mutual inductance measured for the Nb rich film with a thickness of 33 nm. Just like the conventional ac susceptibility, the real part of the mutual inductance (M′) measures the magnetic shielding (diamagnetic property) response generated by super-currents, while the imaginary part (M″) is related to the energy dissipation and losses in the material. In zero magnetic field, the temperature at which diamagnetic shielding currents develop in the superconducting film, |M″| shows a peak indicating an increase in dissipation due to flux flow. However, with increasing magnetic field, the |M″| of the nano-composite films showed very interesting features. An additional peak appeared at slightly lower temperatures which was of lower amplitude and broader as compared to the first peak. The two peaks persisted upto the upper critical field of the film. The appearance of the two peaks in |M″| with magnetic field was also observed for the Cu rich films. Figure 3a,b show the temperature dependence of |M′| and |M″| in different magnetic fields for the film with thickness of 72 nm. However, the second peak at lower temperatures had a higher amplitude than the first peak contrary to the Nb rich films (Fig. 2b). We attribute the two peaks with the intra and inter-granular losses for granular superconductors in line with the observations and discussions related to high T_c_ Cuprate superconducting thin films^32–34^. The peak at higher temperatures, T_ph_ (close to T_c_ or the temperature where the diamagnetic shielding response develops in the films) can be associated with the increase in dissipation due to the movement of intra-granular vortices within the superconducting Nb grains. The peak at lower temperatures, T_pl_ can be attributed to dissipation arising due to breaking of inter-granular Josephson coupling leading to flux flow in the grain boundaries and Cu grains. For Nb rich films, the intra-granular component is expected to be larger due to the presence of a larger number of Nb grains. For the Cu rich film with lesser number of Nb grains, the inter-granular component dominates leading to greater losses.

Furthermore, to get an estimate of the upper critical fields and hence the coherence lengths of the films, phase diagrams (H vs T) were plotted. Figures 2c and 3c show the phase diagrams for the Nb rich and Cu rich thin films respectively. In both the plots, the black squares denote the temperature at which the diamagnetic shielding response of the films show a drop (as obtained from the |M′| vs T plot for different magnetic fields). We also show the phase line obtained from the two peaks at temperatures T_pl_ (blue triangles) and T_ph_ (red circles) of the |M″| vs T plots. All three phase lines for all the Nb–Cu nano-composite films (Nb rich as well as Cu rich) showed a linear variation which can be explained on the basis of models for 3D granular superconductors^11^. To obtain the upper critical fields, we used the WHH theory applicable for dirty granular superconductors^35^. Since, ${H}_{c2}\left(0\right)=0.69 {T}_{c}{\left(\frac{d{H}_{C2}}{dT}\right)}_{{T}_{c}}$, H_c2_(0) was about 2.28 T for the 33 nm thick Nb rich film and 1.52 T for the 72 nm thick Cu rich film giving the coherence lengths of 12 and 15 nm respectively. This shows that the Nb–Cu films studied here are in the 3D limit with the thickness much greater than the coherence length. However, the Nb grains in the films have sizes comparable with the coherence length. This indicates that QSE in the individual Nb grains may play a role in influencing superconductivity^15^. Similarly, SPE due to the proximity of the small Cu grains to the nano-grains of Nb may affect the properties of the films, depending on the cleanliness of the Nb–Cu interfaces^16^. Besides, as discussed previously phase fluctuations in the random array of SNS Josephson junctions might also influence the superconducting properties just like it did in 2D granular films having an array of SIS junctions^19–22^. To see which of the mechanisms play the dominant role, we analyzed the two-coil mutual inductance data for finite films to obtain the superfluid stiffness (J_S_) and the penetration depth (λ).

**Figure 4:**
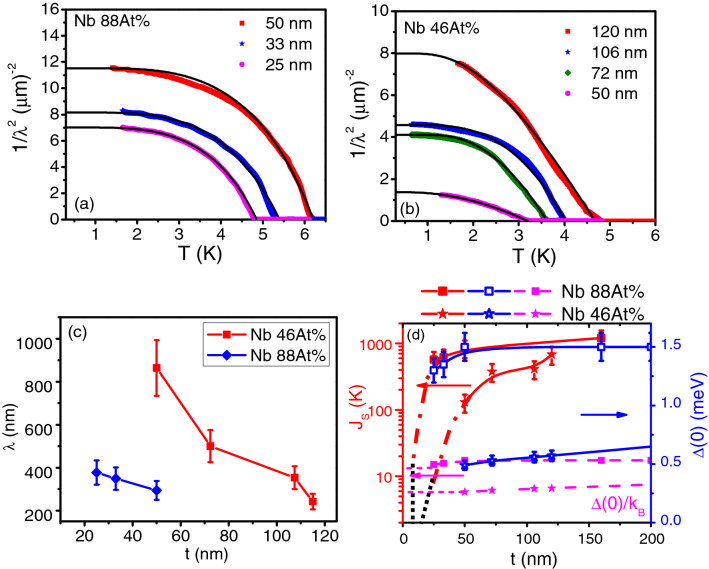
(**a**) Plot of 1/λ^2^ with temperature (T) for Nb rich (Nb 88 at%) films with different thicknesses of 50 nm (red squares), 33 nm (blue stars) and 25 nm (magenta circles). (**b**) Plot of 1/λ^2^ with temperature (T) for Cu rich (Nb 46 at%) films with different thicknesses of 120 nm (red squares), 106 nm (blue stars), 72 nm (green diamonds) and 50 nm (magenta circles). For all curves in (**a**,**b**) the solid symbols are obtained by analyzing the experimental (M′ and M″ vs T) data based on the theoretical model (see text), while the solid black lines indicate the BCS fits based on the relation given in the text. (**c**) Variation of the penetration depth (λ) with thickness (t) for Nb rich (Nb 88 at%) films (blue diamonds) and for the Cu rich (Nb 46 at%) films (red squares). (**d**) Variation of superfluid stiffness (J_S_) and superconducting energy gap ∆(0) as a function of film thickness (t). The scale for the superfluid stiffness (J_S_) is shown on the left and that for ∆(0) is shown on the right. J_S_ is indicated by red squares and red stars for the Nb rich and Cu rich films respectively. ∆(0) is shown by blue squares and blue stars for the Nb rich and Cu rich films respectively. ∆(0)/k_B_ (scale on the left) is also represented for each film of Nb rich (magenta squares) and Cu rich (magenta stars). The black dashed lines are extrapolation of the J_S_ data for both the compositions to see where is crosses the ∆(0)/k_B_ lines.

Figure 4a, b show the variation of the 1/λ^2^ ($\propto {n}_{S} \propto {J}_{S}$, where n_S_ is the superfluid density and J_S_ is the superfluid stiffness) with temperature for different thicknesses for the Nb rich and Cu rich films respectively (This was obtained by analyzing the experimental M′ and M′′ vs T data for each film based on the theoretical model of Refs.^24,25^). As expected, with a decrease in film thickness, 1/λ^2^ decreases^36^ for both the compositions. Moreover, with an increase in Cu content, of comparable thickness, J_S_ decreased indicating that the presence of larger number of SNS junctions has a lower energy cost to twist the phase. The temperature variation of 1/λ^2^ was fitted using the BCS relation in the dirty limit^1^ applicable for granular superconductors with small grains given by: $\frac{{\lambda }^{-2}\left(T\right)}{{\lambda }^{-2}(0)}= \frac{\Delta (T)}{\Delta (0)}\tanh\left[\frac{\Delta (T)}{2{k}_{B}T}\right]$, where ∆(0) is a fitting parameter. The fits were reasonably good over the entire temperature range for all the films. Interestingly, no linear dependence of λ was observed with temperature which has been reported as a signature for the presence of thermal phase fluctuations in 3D films^8,9^. λ for the Nb rich films varied between 300 to 400 nm with decrease in thickness from 50 to 25 nm. However, it increased substantially for the Cu rich films, reaching to 800 nm for the film with thickness of 50 nm (see Fig. 4c). Besides, ∆(0) obtained from the fits were close to the BCS value of ∆(0) ~ 1.76 k_B_T_c_ (Blue squares for the films with Nb 88 at% and blue stars for the films with Nb 46 at% in Fig. 4d, where the right-hand scale is used for ∆(0)). Next, using ${J}_{S}= \frac{{\hbar}^{2}\xi {n}_{S}}{4{m}^{*}}$ and the values of ξ and λ determined from the experiments, we calculated J_S_ for the films. In Fig. 4d we plot J_S_ in K (shown in log scale) as a function of the thickness of the films. The red squares are for the films with Nb 88 at% and red stars are for the films with Nb 46 at%. The left-hand scale is used to show J_S_. In the same figure, we have plotted ∆(0)/k_B_ (in K) shown as magenta points (squares and stars for the films with Nb content as 88 at% and 46 at% respectively). We see that J_S_ > > ∆(0)/k_B_ for both the compositions for the thinnest films studied indicating that phase fluctuations do not play any role in superconductivity in the Nb–Cu 3D granular films down to 50 nm for a film with Nb 46 at%. Furthermore, if we extrapolate the variation of J_S_ to lower thickness (black dashed line in Fig. 4d), we see that J_S_ ~ ∆(0)/k_B_ for films with thicknesses < 5 nm for the Nb rich nano-composites while for the Cu rich films it becomes comparable at thickness < 25 nm i.e. very close to the 2D regime similar to the granular Al films studied previously^19–22^. This is indicative that for Nb–Cu films with higher Cu content (> 60 at%), it is possible to see the effect of phase fluctuations on superconducting properties even for relatively thick films (~ 20–30 nm) i.e. in 3D. However, to prove this statement, one needs to be able to measure the superfluid stiffness to very low temperatures (~ 0.1 K) since it is expected that for this film thickness and Cu content, the T_c_ will also be appreciably decreased.

Now, if phase fluctuations are ruled out for the observed variation of the superconducting properties with change in Cu content in the Nb–Cu films, the next pertinent question which follows is, which is the other dominant mechanism influencing superconductivity in these nano-composite films? It is known that QSE arises due to the discretization of the energy levels in small particles. For BCS superconductors, where ${T}_{c}=1.14{\theta }_{D}\mathrm{exp}\left(-\frac{1}{N\left(0\right)V}\right)$ (here, θ_D_ is the Debye temperature, N(0) is the density of states at the Fermi level and V is the electron–phonon interaction potential), as N(0) decreases, T_c_ is known to decrease with decreasing particle size^15^*.* As discussed in our previous report on thick Nb–Cu films, QSE was seen to influence the T_c_^ON^ (the onset of the superconducting transition in the R-T measurements) and the upper critical field, H_C2_ (determined from the phase diagram plotted using the T_c_^ON^ in R-T measurements in magnetic fields)^31^. Now as the films are above the percolation threshold, and T_c_ is very close to T_c_^ON^, one possibility is that T_c_ is also affected by the small size of the Nb grains. It is worthwhile to note that with doubling the Cu content in the films, the average particle size of Nb was seen to decrease from 16 to 8 nm which could lead to a drop in T_c_ by 2 K (as seen from previous studies in nanocrystalline Nb films^15^). While this scenario seems quite plausible, looking at the microstructure of the films, the presence of Cu nano-particles (of sizes less than the coherence length) in close proximity to the Nb nano-particles cannot be totally neglected in understanding the variation of T_c_ with composition. There are very few reports in the literature which discusses the variation of the T_c_ of such nano-composite granular films on the basis of the superconducting proximity effect (SPE). In a couple of reports, T_c_ has been observed to vary exponentially as a function of the ratio of the volume fractions of the superconducting and normal metal components in the films^16^. Using this model, if we estimate the T_c_ of the Nb–Cu films of Nb 88 at% and Nb 46 at%, they do not seem to follow our results (say a thick film of both concentrations should show a T_c_ ~ 7.0 and ~ 0.7 K respectively)^37,38^. Clearly, the very simplistic model of Ref.^16^ does not seem to explain our results. Another aspect regarding the microstructure of the films which is worth considering is the presence of rough interfaces or dis-ordered intergranular region of the Nb grains which is well known for sputter grown Nb nanocrystalline films^39^. This would lead to increased diffuse scattering at the grain boundaries of the superconducting grains which might reduce the effect of SPE. Thus, we conjecture that though it is difficult to quantitatively estimate the dominant mechanism of superconductivity in the nano-composite films consisting of random network of SNS junctions, it is most likely that an interplay of QSE and SPE affects T_c_ in these films.

## Conclusions

In conclusion, we have studied a random network of SNS nano-junctions forming 3D granular films of Nb-Cu for two distinct compositions (Nb rich with Nb 88 at% and Cu rich with Nb 46 at%) with varying film thickness. T_c_ was observed to decrease with both decreasing thickness and decreasing Nb content in the films. However, for all films studied, the nano-grains of Nb and Cu remained well connected leading to low values of the film resistivity (~ μohm-cm). Inspite of this, in the presence of the external magnetic field, the imaginary component of the mutual inductance (M″) which measured the losses in the films showed two peaks at two temperatures (T_ph_ and T_pl_) corresponding to the intra and inter-granular pair breaking effect of the magnetic field. With increase in Cu content in the films (Nb 46 at%), the inter-granular contribution to the losses dominated over the intra-granular contribution suggesting an increase in the S–N–S junctions. Furthermore, the coherence length (ξ) estimated from these measurements showed that all the films studied in the present work were in the 3D limit (as d > ξ). The penetration depth (λ) of the films was measured using the two-coil mutual inductance measurements which were also used to compute the superfluid stiffness (J_S_) of the films. λ was observed to expectedly increase with decreasing film thickness for a particular composition of the films. Moreover, it was also seen to increase with increasing Cu content implying a decrease in J_S_. However, this decrease was not sufficient to lead to phase fluctuations in the films with the lowest thickness probed in this work as it remained an order of magnitude higher than the pair breaking energy, ∆ or the superconducting energy gap (obtained from the analysis of the temperature dependence of the mutual inductance data). In addition, the temperature variation of J_S_ followed the standard BCS variation over the entire temperature range ruling out any role of thermal phase fluctuations. Thus, in 3D granular nano-composite films consisting of random network of SNS junctions, phase fluctuations are overpowered by an interplay of QSE and SPE in controlling superconductivity. Furthermore, our results show that to really see the effect of phase fluctuations on superconductivity in films with random network of superconductor and normal metal nanoparticles, one needs to probe close to the 3D-2D cross-over regime similar to granular Al and unlike homogenously dis-ordered NbN films.

## Data Availability

The datasets generated during and/or analysed during the current study are available from the corresponding author on reasonable request.
